# PP83 Building Resilient Capacity For The Diagnosis Of Cardiac Pathologies Through Telemedicine: Pilot Study

**DOI:** 10.1017/S0266462324002526

**Published:** 2025-01-07

**Authors:** Pedro Galvan, José Ortellado, Ronald Rivas, Benicio Grossling, Enrique Hilario

## Abstract

**Introduction:**

The evolution of technology in medicine and information and communication technology (ICT) has been an important step to introduce innovative/resilient specialized health services. The emergence of telemedicine offers opportunities to enhance specialized diagnosis services. This study has evaluated the feasibility of using tele-Holter and tele-ABPM (ambulatory blood pressure monitoring) to build a resilient diagnosis network for cardiac pathologies in Paraguayan remote public hospitals.

**Methods:**

This observational descriptive multicenter feasibility study is based on a telemedicine-driven approach for specialized cardiac diagnostic services using Holter and ABPM devices in remote and underserved public hospitals in Paraguay. A telemedicine platform was used to send records of Holter and ABPM devices from the remote hospitals to the cardiologist to screen cardiac pathologies.

**Results:**

During the pilot study, 52 cardiac diagnostic tests were carried out using the tele-Holter and tele-ABPM approach in 10 regional hospitals countrywide. Cardiac diagnosis was performed in 24 patients using Holter and 28 patients using ABPM. The most frequent findings using tele-Holter were normal (91.6%), not sustained ventricular tachycardia (4.2%), and atrial fibrillation (4.2%). Regarding tele-ABPM, the diagnoses performed were arterial hypertension (50.0%), uncontrolled arterial hypertension (40.0%), and normal (10.0%). Overall, an average of 90.0 percent of diagnosed patients suffered high blood pressure and 8.4 percent suffered heart disorder.

**Conclusions:**

According to our results, the use of a telemedicine-driven approach to build a resilient diagnosis network for cardiac pathologies in remote underserved public hospitals in Paraguay is feasible. A widespread use-assessment should be analyzed before this tool is adopted.

